# CONNECT Powered by BRAIN: Harnessing AI-Driven Behavior Analysis to Foster Person-Centered Dementia Programming

**DOI:** 10.1093/geroni/igaf122.3738

**Published:** 2025-12-31

**Authors:** Michael Skrajner, Gregg Gorzelle, John Zeisel, Sucheendra Palaniappan, Vivek Saxena

**Affiliations:** Hopeful Aging, Winchester, Massachusetts, United States; Hopeful Aging, Winchester, Massachusetts, United States; Hopeful Aging, Winchester, Massachusetts, United States; Vikasietum Tecknology LLC, Plano, Texas, United States; Vikasietum Tecknology LLC, Plano, Texas, United States

## Abstract

Person-centered activity programming can reduce responsive behaviors and support meaning and purpose for persons living with dementia (PLwD; Chenoweth et al., 2009; Lee et al., 2012). Yet tailoring activities to an individual’s cognitive level and interests typically depends on front-line staff, a time-intensive process often infeasible in long-term care. Hopeful Aging, through an NIA-funded SBIR Phase II project, is developing CONNECT powered by BRAIN, an AI-driven platform that personalizes and streamlines activity programming. The system draws from a growing, curated library of over 1,000 dementia-appropriate activities across 12 types. Based on a PLwD’s cognitive profile and interests, the platform generates personalized recommendations, each scored for fit (0–100). To minimize decision fatigue, the top seven recommendations appear on the first screen. Recommendations then adapt dynamically through AI-based behavioral engagement analysis derived from session recordings (with consent/assent). Engagement is quantified using behavioral markers—verbal communication, visual attention, pleasure, and hand and head gestures—and compared against the individual’s evolving baseline to refine subsequent suggestions. In Phase I, 22 participants (M age=83.7; 82% female; 73% Caucasian) tested an alpha prototype. A subsequent Model Training Study in the ongoing Phase II project with 26 participants (M age=84.5; 76% female; 68% Caucasian) refined behavioral analyses. Post-training F1 values were: Verbal Communication=90%; Visual Attention=87%; Pleasure=86%; Hand Gestures=82%; Head Gestures=76%, with a mean improvement of + 9%. Findings demonstrate the feasibility of automated AI engagement analyses for adaptive, person-centered activity programming. A forthcoming cluster-randomized trial will examine impacts on quality of life, responsive behaviors, and depression.

